# Cardiac metabolic changes on ^18^F‐positron emission tomography after thoracic radiotherapy predict for overall survival in esophageal cancer patients

**DOI:** 10.1002/acm2.13552

**Published:** 2022-03-04

**Authors:** Sara J Zakem, Bernard Jones, Richard Castillo, Edward Castillo, Moyed Miften, Karyn A Goodman, Tracey Schefter, Jeffrey Olsen, Yevgeniy Vinogradskiy

**Affiliations:** ^1^ Department of Radiation Oncology University of Washington Seattle Washington USA; ^2^ Department of Radiation Oncology University of Colorado School of Medicine Aurora Colorado USA; ^3^ Department of Radiation Oncology Emory University Atlanta Georgia USA; ^4^ Department of Radiation Oncology Beaumont Health Royal Oak Michigan USA; ^5^ Department of Radiation Oncology Mount Sinai New York New York USA; ^6^ Department of Radiation Oncology Thomas Jefferson University Philadelphia Pennsylvania USA

**Keywords:** cancer, esophageal, functional imaging, heart dose, PET, radiation

## Abstract

**Purpose:**

Heart doses have been shown to be predictive of cardiac toxicity and overall survival (OS) for esophageal cancer patients. There is potential for functional imaging to provide valuable cardiac information. The purpose of this study was to evaluate the cardiac metabolic dose–response using ^18^F‐deoxyglucose (FDG)–PET and to assess whether standard uptake value (SUV) changes in the heart were predictive of OS.

**Methods:**

Fifty‐one patients with esophageal cancer treated with radiation who underwent pre‐ and post‐treatment FDG–PET scans were retrospectively evaluated. Pre‐ and post‐treatment PET‐scans were rigidly registered to the planning CT for each patient. Pre‐treatment to post‐treatment absolute mean SUV (SUVmean) changes in the heart were calculated to assess dose–response. A dose–response curve was generated by binning each voxel in the heart into 10 Gy dose‐bins and analyzing the SUVmean changes in each dose‐bin. Multivariate cox proportional hazard models were used to assess whether pre‐to‐post treatment cardiac SUVmean changes predicted for OS.

**Results:**

The cardiac dose–response curve demonstrated a trend of increasing cardiac SUV changes as a function of dose with an average increase of 0.044 SUV for every 10 Gy dose bin. In multivariate analysis, disease stage and SUVmean change in the heart were predictive (*p* < 0.05) for OS.

**Conclusions:**

Changes in pre‐ to post‐treatment cardiac SUV were predictive of OS with patients having a higher pre‐ to post‐treatment cardiac SUV change surviving longer.

## 1 INTRODUCTION

Radiation therapy plays an essential role in the management of esophageal cancer.^1^ Radiation treatment planning for esophageal cancer patients remains challenging due to the geometric proximity of normal tissue organs relative to the disease. In particular, the heart is considered an important dose‐limiting organ when designing radiation treatment plans for esophageal cancer patients. Recent studies have highlighted the importance of managing doses to the heart.^2^, ^3^ Radiation doses to the heart have been shown to predict for cardiac toxicity,^3^ cardiac death,^4^ and overall survival (OS)^5^, ^6^ for patients receiving thoracic radiotherapy for either esophageal or lung cancer.

Although dosimetric parameters remain an important factor in predicting for toxicity and disease control,^7^, ^8^, ^9^, ^10^ studies have shown that incorporating functional imaging can improve the ability to predict for clinical outcomes.^11^ Thorwarth et al. demonstrated that hypoxia‐based positron emission tomography (PET) can predict for loco‐regional failure^12^ in head and neck cancer patients. In lung cancer patients, Aerts et al.^13^ showed that PET‐computed tomography (PET‐CT) can be used to aid in the prediction of OS. In terms of normal tissue toxicity, studies have highlighted the ability of functional lung imaging to predict for thoracic toxicity^14^ and information gathered from PET to predict for xerostomia in head and neck cancer patients.^15^ With respect to cardiac evaluation, researchers have been able to use cardiac functional imaging to characterize a dose–response^16^, ^17^, ^18^, ^19^ for various disease sites including breast and lung cancer. There is potential for functional imaging to provide valuable cardiac information for esophageal cancer patients treated with radiation therapy.

^18^F‐deoxyglucose (FDG)–PET plays an essential role in the management of esophageal cancer.^1^ FDG–PET is used for disease diagnosis, staging, and monitoring of disease response to therapy^20^ for esophageal cancer patients. Although FDG–PET is primarily considered a diagnostic and tumor staging tool, studies have shown that FDG–PET can be used to evaluate the radiation response of normal tissues^21^, ^22^, ^23^ and provide a diagnostic marker of cardiac inflammation.^22^ PET therefore has the potential to provide a functional imaging cardiac biomarker for esophageal cancer patients using imaging that is acquired as standard of care without burdening the patient with an extra imaging procedure. The purpose of this study was to evaluate the cardiac metabolic dose–response using FDG–PET for esophageal cancer patients and assess whether SUV changes in the heart were predictive of OS.

## 2 METHODS

### 2.1 Study population

Patients with esophageal cancer treated with radiation therapy at a single University health system from 2012 to 2019 were retrospectively reviewed. Inclusion criteria for the current study included a pathologically confirmed diagnosis of esophageal cancer, treatment with radiation therapy, and the availability of both pre‐ and post‐treatment FDG–PET scans. All data were collected under an institutional review board approved protocol for retrospective data analysis. Fifty‐one patients met the study inclusion criteria and were included in the current analysis. All patients had pre‐ and post‐radiotherapy PET‐CT acquired as standard of care for disease diagnosis, disease staging, and to evaluate disease response to therapy.

### 2.2 Cardiac PET evaluation

For each patient, the attenuation‐corrected pre‐treatment and post‐treatment PET‐CT scans were rigidly registered to the treatment planning CT. Each registration was performed using MIM Software (Cleveland, OH)^24^ with a region of interest that included the heart, mediastinum, and lungs. Each registration was manually reviewed with a focus on the cardiac region and adjusted manually if necessary. The heart contours delineated by the physician that were used for the patient's clinical treatment plan were used for the current analysis. The mean standardized uptake value (SUV, SUVmean) and maximum SUV (SUVmax) were calculated for both the pre‐ and post‐treatment heart contours. The SUVmean was computed for each patient by averaging all SUV values within voxels defined by the heart contour. The SUVmax was calculated by taking the maximum SUV value among all voxels within the heart contour for each patient. Population‐based SUVmean and SUVmax values are reported in the results section as mean ± standard deviation.

A population‐based cardiac dose–response curve was generated using pre‐ to post‐treatment SUV changes in the heart. The first step was to bin voxels in the heart contour for each patient into 10 Gy dose‐bins from 0 Gy to 60 Gy (0–10 Gy, 10–20 Gy, 20–30 Gy, 30–40 Gy, 40–50 Gy, and 50–60 Gy). The average SUVmean and SUVmax were then computed in each bin and the absolute difference between SUVmean and SUVmax from pre‐ to post‐treatment was calculated for each patient. To generate a population‐based dose–response curve the pre‐ to post‐treatment SUVmean and SUVmax differences were averaged for the entire patient cohort. A positive SUVmean or SUVmax change indicated post‐treatment cardiac SUV values were greater than pre‐treatment values, while a negative SUVmean or SUVmax change indicated decreased post‐treatment cardiac SUV when compared to pre‐treatment. The results are presented as a dose–response curve (SUV change plotted as a function of dose‐bin) with linear regression used to evaluate for significance.

Pre‐ to post‐treatment changes in cardiac SUV were compared for patients treated with different treatment techniques including rotational intensity modulated radiation therapy (IMRT), IMRT, and three‐dimensional conformal radiation therapy (3D‐CRT). Comparisons in SUV changes were done using a *t*‐test.

### 2.3 Overall survival assessment

The study evaluated whether overall cardiac changes in SUVmean or SUVmax were predictive of OS. Survival was calculated as time between disease diagnosis and death or last known follow‐up. In order to perform a complete survival analysis, additional co‐variates were considered including chronic obstructive pulmonary disease (COPD) status, histology (squamous cell carcinoma versus adenocarcinoma), whether an esophagectomy was performed, Karnofsky performance status (KPS), clinical disease stage, and heart V40Gy^5^ (volume of heart receiving ≥ 40 Gy). Multivariate Cox proportional hazards regression analysis was used to evaluate the impact of SUV, patient, clinical, and treatment factors on OS. Kaplan–Meier survival curves are presented for two methods of dividing the study population according to SUV thresholds: a SUVmean threshold of zero (indicating whether the post‐treatment cardiac SUV were larger or smaller than the pre‐treatment cardiac SUV) and an optimally determined SUVmean threshold using previously described methods.^25^

## 3 RESULTS

Between 2012 and 2019, a total of 158 patients with a pathologically confirmed diagnosis of esophageal cancer who were candidates for radiation therapy were seen in the department of radiation oncology at our institution. Of these, 51 patients met inclusion criteria for the present study which included the availability of pre‐ and post‐treatment PET scans as well as radiation treatment planning data. Patient, clinical, and radiation treatment factors for the 51‐patient cohort are summarized in Table 1. The median age was 67 years old, the majority of patients (71%) were current or former smokers, and the median KPS was 80. The most common diagnosis was adenocarcinoma (74%) with stage III disease (43%). The majority (92%) of patients received concurrent chemotherapy and 45% of patients underwent an esophagectomy. The median prescription dose was 50.4 Gy (range 30–60 Gy) delivered in 25 fractions (range 10–38 fractions). The mean heart dose was 18.0 Gy with a range of 0.1–30.1 Gy) and the median V40Gy was 5.9% (range 0.0%–18.4%). The median time between the follow‐up PET‐CT scan and the end of radiotherapy was 56 days (range 3‐692 days).

**TABLE 1:** Patient, clinical, and radiation parameters of study cohort

**Parameter**	**Number (%) or median (range)**
Number of patients	51
Gender	
Female	17 (33)
Male	34 (67)
Age	67 (40–89)
COPD	
Yes	7 (14)
No	44 (86)
Smoking status	
Non‐smoker	15 (29)
Current smoker	7 (14)
Former smoker	29 (57)
KPS Index	80 (60–90)
Type of esophageal cancer	
Adenocarcinoma	38 (74)
Squamous cell carcinoma	13 (26)
Stage	
II	15 (29)
III	22 (43)
IV	8 (16)
Unknown	6 (12)
Surgical Status	
Esophagectomy performed	23 (45)
Esophagectomy not performed	28 (55)
Concurrent Chemotherapy Status	
Concurrent Chemotherapy administered	47 (92)
Concurrent Chemotherapy not administered	4 (8)
Fractionation	
Total dose (Gy)	50.4 (30–60)
Number of fractions	25 (10–38)
Treatment technique	
3D‐CRT	6 (12)
Static IMRT	9 (18)
Rotational IMRT	36 (70)
Heart doses	
Mean heart dose (Gy)	18.0 (0.1–30.1)
V5 (%)	85.8 (0.0–100.0)
V30 (%)	15.5 (0.0–43.0)
V40 (%)	5.9 (0.0–18.4)
V45 (%)	3.1 (0.0–14.3)
Time between last radiation treatment and post‐treatment PET scan (days)	56 (3–692)

Abbreviations: COPD, Chronic obstructive pulmonary disease; 3D‐CRT, 3‐dimensional conformal radiation therapy; Heart V5‐40 = percentage of heart receiving ≥5–40 Gy; IMRT, intensity‐modulated radiation therapy; KPS, Karnofsky performance status.

**FIGURE 1:**
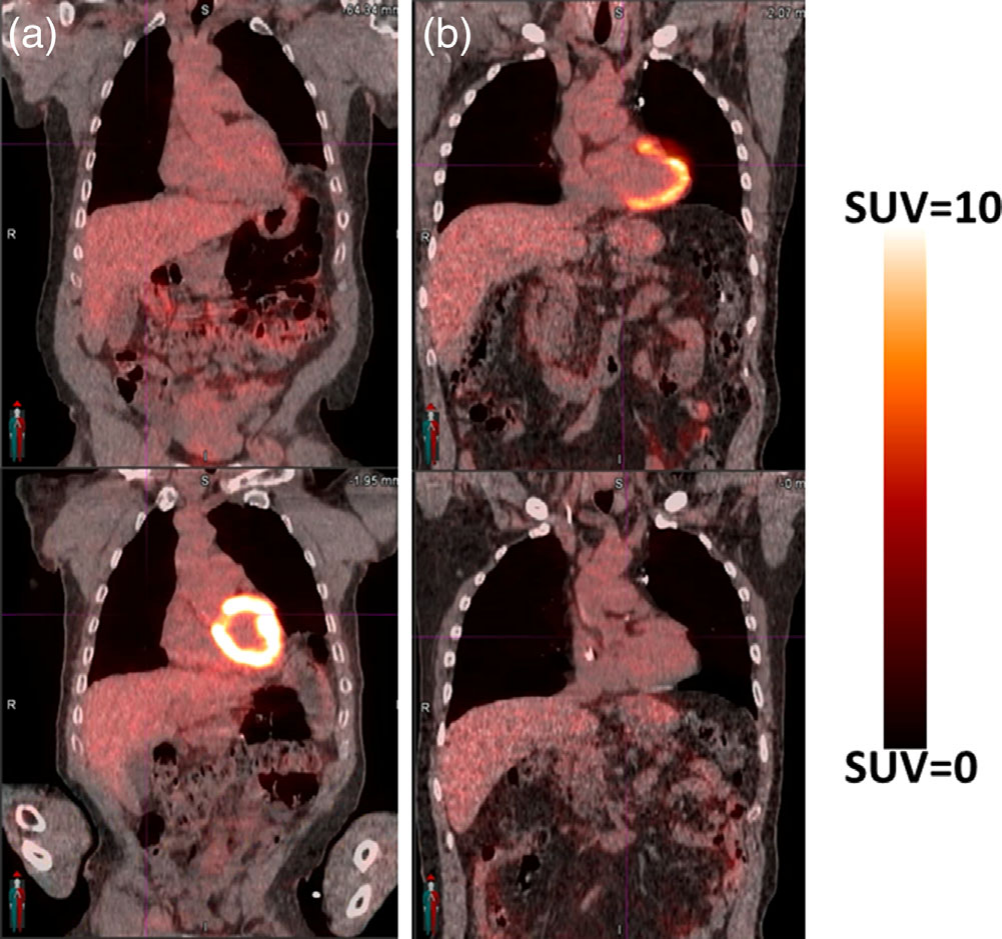
Patient examples of a patient with (a) increased post‐treatment cardiac SUV compared to pre‐treatment cardiac SUV and (b) decreased post‐treatment cardiac SUV when compared to pre‐treatment. SUV, standard uptake value

**FIGURE 2:**
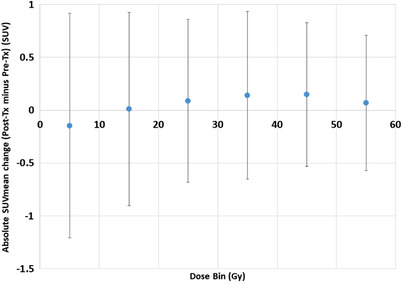
Dose–response curve showing the absolute SUVmean change as a function of dose‐bin. Error‐bars are presented as mean ± standard deviation. SUV, standard uptake value; post‐tx, post‐treatment

The average SUVmean values for the entire heart for the pre‐treatment and post‐treatment PET scans were 1.98 ± 0.70 and 1.98 ± 0.90, respectively. The average SUVmax values for the entire heart for the pre‐treatment and post‐treatment PET scans were 5.69 ± 3.56 and 6.02 ± 4.63, respectively. Representative patient examples of a patient with a large decrease in SUV from pre‐ to post‐treatment and a large increase in SUV from pre‐ to post‐treatment are presented in Figure 1. A cardiac dose–response curve showing the change in pre‐ to post‐treatment SUVmean is presented in Figure 2.

The number of patients contributing to the 0–10 Gy, 10–20 Gy, 20–30 Gy, 40–50 Gy, and 50–60 Gy dose‐bins, were 46 (91%), 44 (86%), 44 (84%), 42 (82%), 38 (75%), and 28 (55%), respectively. The curve displays an overall trend of increasing SUVmean as a function of dose‐bin (linear regression *p* = 0.083) with an average increase of 0.044 SUV for every 10 Gy dose bin. The average pre‐ to post‐treatment absolute SUVmax change values were 0.23, 0.07, 0.36, 0.23, 0.24, and 0.38 for the 0–10 Gy, 10–20 Gy, 20–30 Gy, 40–50 Gy, and 50–60 Gy dose‐bins, respectively. Similar to the SUVmean trends, the pre‐ to post‐treatment cardiac SUVmax changes showed an overall trend of increasing SUV as a function of dose‐bin (linear regression *p* = 0.256) with a slope of 0.032 SUV increase for every 10 Gy.

Pre‐ to post‐treatment SUV changes were compared among different treatment planning techniques (rotational IMRT, IMRT, and 3D‐CRT). The average SUV changes were 0.04 ± 0.72, 0.25 ± 1.07, −0.65 ± 0.70, for rotational IMRT, IMRT, and 3D‐CRT, respectively. The differences between the SUV changes between the IMRT and 3D‐CRT group were significant (*p* = 0.03); all other comparison produced *p*‐values > 0.09.

Patients were followed for a median of 580 days (range 127–2164 days) with 23 of 51 patients alive at last follow‐up. In multivariate analysis (Table 2), disease stage and SUV changes (both SUVmean and SUVmax changes [only one SUV change factor was included in the multivariate model at a time to avoid collinearity]) were predictive (*p* < 0.05) for OS.

**TABLE 2:** Parameters predictive of overall survival using the multivariate Cox Model

Covariate	HR (95% CI)	*p*
Age	1.0013 (0.969–1.060)	0.560
COPD status (binary: COPD not present vs. COPD present)	0.241 (0.041–1.421)	0.116
Histology (squamous cell carcinomas vs. adenocarcinomas)	0.544 (0.172–1.729)	0.303
Esophagostomy done (binary)	0.478 (0.161–1.425)	0.185
KPS (80 vs. 90)	1.239 (0.464–3.305)	0.669
Clinical Stage 2 (binary: stage 2 vs. stage 3)	0.242 (0.070–0.834)	0.025
Clinical Stage 4 (binary: stage 4 vs. stage 3)	0.081 (0.263–2.494)	0.713
Heart V40 (continuous)	1.047 (0.957–1.144)	0.314
Change (increase) in SUV_Mean_ (continuous)	0.504 (0.275–0.929)	0.028
Change (increase) in SUV_Max_ (continuous)	0.893 (0.801–0.996)	0.042
Treatment technique (rotational IMRT vs. IMRT/3D‐CRT)	1.640 (0.416–6.472)	0.480
Treatment technique (IMRT vs. rotational IMRT/3D‐CRT)	0.549 (0.154–1.953)	0.354

Abbreviation: Change in SUVmean, pre‐treatment to post‐treatment changes in the average cardiac standardized uptake values; COPD, chronic obstructive pulmonary disease ; 3D‐CRT, three‐dimensional conformal radiation therapy; Heart V40, percentage of heart receiving ≥40 Gy; IMRT, intensity‐modulated radiation therapy; KPS, Karnofsky performance status.

**FIGURE 3:**
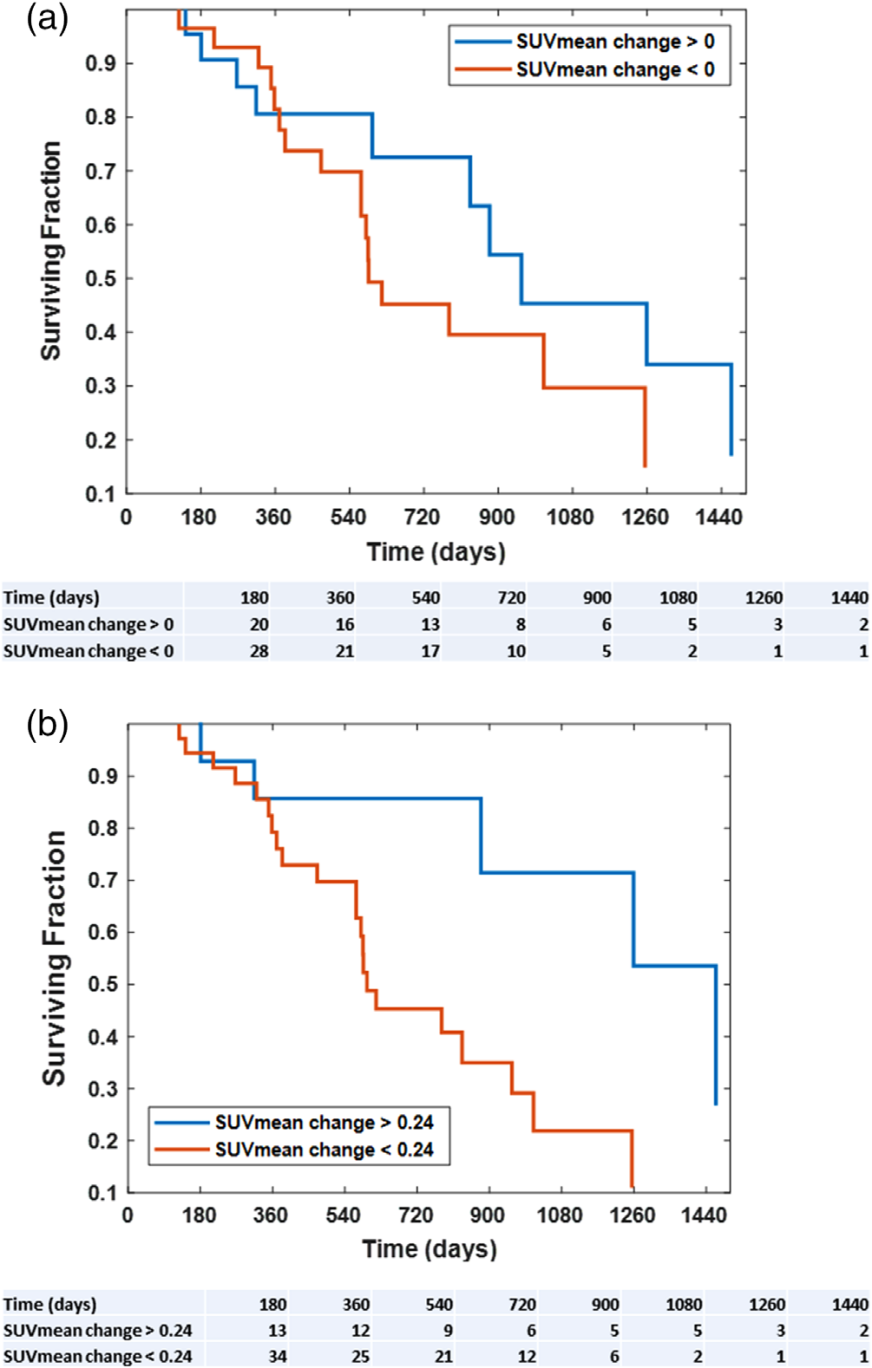
Survival curves presented as a function of SUVmean change. (a) Schematic presents survival curves with patients grouped according to a SUVmean change threshold of zero (*p* = 0.066). (b) Schematic presents survival with patients grouped using an optimally determined threshold of a SUVmean change of 0.24 (*p* = 0.036)

The hazard ratio for the pre‐ to post‐treatment SUVmean change was 0.504 (95% confidence interval of 0.275–0.929) (Table 2). Figure 3 presents Kaplan–Meier survival curves with patients grouped according to a SUVmean change threshold. An optimally determined threshold was able to significantly separate patient cohorts (*p* = 0.036). A Kaplan–Meier curve separating patients using a threshold of zero (separating patients according to whether they had an increase or decrease in cardiac SUV from pre‐ to post‐treatment) was not statistically significant (*p* = 0.066).

## 4 DISCUSSION

Our data indicate that changes in the pre‐ to post‐treatment FDG–PET cardiac SUV were predictive of OS (*p* = 0.028). Specifically, patients who had an increase in cardiac SUV post‐treatment when compared to pre‐treatment had significantly improved OS (Figure 3a,b). FDG–PET scans are frequently acquired as standard of care for esophageal cancer patients for disease staging and to evaluate disease response to treatment. Our data demonstrate the potential of cardiac functional imaging changes evaluated in the pre‐ and post‐treatment FDG–PET to provide an early predictor of clinical outcomes. The presented cardiac functional imaging data for esophageal cancer patients are in line with results that have been reported for lung cancer patients.^26^ Vinogradskiy et al.^26^ evaluated PET‐based SUV changes in the heart for lung cancer patients who underwent chemo‐radiation and found similar trends. Specifically, the study in lung cancer patients noted that pre‐ to post‐treatment PET changes were predictive of OS and that an increase in SUV from pre‐ to post‐treatment corresponded with improved OS.^26^ Taken together, these studies indicate the potential of pre‐ to post‐treatment SUV changes in the heart to be used as a predictive biomarker for patients receiving radiotherapy to the thorax.

Although the results were not significant using linear regression, our data revealed an overall trend of increasing SUV cardiac changes as a function of dose. The dose–response data are in line with cardiac imaging results reported for esophageal cancer patients,^27^, ^28^ lung cancer patients,^21^, ^26^, ^29^ and head and neck cancer patients.^30^ Jingu et al.^28^ showed focal increased SUV uptake (using FDG–PET scans) in the myocardium after radiotherapy for esophageal cancer patients and Takanami et al.^27^ demonstrated correlations between radiation dose to the heart and myocardium metabolism (using single photon emission computed tomography (CT) imaging). For advanced‐stage lung cancer patients, the data presented by Vinogradskiy et al.^26^ showed increasing FDG uptake in the heart as a function of dose, while the study presented by Guerrero et al.^21^ showed increasing FDG signal in normal lung as a function of dose. Evans et al.^31^ demonstrated increased SUV with increasing dose for early‐stage lung cancer patients treated with stereotactic body radiation therapy. The data presented in the literature along with the currently presented results underscore the capability of functional imaging to characterize a dose–response in normal tissues.

Although FDG–PET is typically used to assess the disease, studies have underlined the capability of FDG–PET to provide clinically relevant information for normal tissues.^21^, ^22^, ^26^, ^28^ FDG–PET protocols are available that can be used to diagnose cardiac inflammatory conditions^22^ (although it should be noted that fasting and imaging protocols can differ between FDG–PET oncologic applications and cardiac inflammatory imaging applications) and to evaluate metabolic viability of the myocardium.^32^ Case reports have shown increased cardiac FDG uptake in the heart after radiotherapy.^33^, ^34^ Studies have quantified the ability of FDG–PET to elucidate a dose–response^21^, ^26^, ^28^, ^29^, ^30^ and have shown how the functional imaging changes can be predictive of clinical outcomes.^21^, ^26^, ^28^ The current work is hypothesis generating, and if validated in prospective trials, provides data supporting the idea of using pre‐ and post‐treatment FDG–PET scans for early identification for patients with divergent clinical outcomes.

Because the current work was a retrospective study, the post‐treatment PET scans were acquired at variable time points and there were no attempts made to perform the pre‐ and post‐treatment imaging on the same PET‐CT scanner. The fasting protocol used for the PET scans was focused on oncologic applications which differ from fasting protocols used to evaluate cardiac inflammation. Despite the lack of consistent scan timing, scanner selection, and non‐specific fasting protocols, our data were able to demonstrate that cardiac PET changes were predictive of clinical outcomes. A prospective clinical trial which homogenizes scanner selection, scan timing, and fasting protocols will be the focus of future work. A limitation of the current work is the 51‐patient sample‐size. Although 51 patients are in line with previous functional imaging work,^26^ 51 patients are a sub‐optimal number of patients for robust survival statistics. Consistent with previous dose–response work,^21^, ^26^, ^31^, ^35^ the current study used rigid registration to register the pre‐ and post‐treatment PET scans to the treatment planning CT. Although all registrations were manually reviewed, the registration uncertainty may contribute to the uncertainty in the dose–response curve.

## 5 CONCLUSIONS

The current study evaluated pre‐ to post‐treatment cardiac SUV changes for esophageal cancer patients treated with radiation therapy. Changes in pre‐ to post‐treatment cardiac SUV were predictive of OS (*p* = 0.028) with patients having a higher pre‐ to post‐treatment cardiac SUV change likely to survive longer. The presented data highlight the potential of functional imaging changes in the heart to provide an early predictor of cardiac‐based morbidity and mortality for esophageal cancer patients.
